# Credelio® Plus: a novel oral endectocide for dogs

**DOI:** 10.1186/s13071-021-04774-7

**Published:** 2021-05-28

**Authors:** Donato Traversa

**Affiliations:** Faculty of Veterinary Medicine, University Teaching Veterinary Hospital, Località Piano D’Accio Snc, 64100 Teramo, Italy

Dogs are exposed to intestinal or extra-intestinal nematodes and ectoparasites throughout their life, irrespective of their age and lifestyle; at the same time, they are at risk of diseases transmitted by arthropod vectors. The effective control of internal and external parasites is crucial for the health and welfare of companion dogs and their owners, as many parasites may also be zoonotic (e.g. roundworms and hookworms) or transmit severe diseases, as in the case of fleas and ticks. For these reasons, the availability of safe and efficacious broad-spectrum endectocides in canine veterinary practice is fundamental for veterinarians and owners.

Credelio Plus is a new and novel oral endectocide intended for use in dogs, encompassing the well-known safety and efficacy of milbemycin oxime with the innovative, safe and efficacious *per os* administration of lotilaner. The macrocyclic lactone milbemycin oxime has been available commercially for over 20 years in multiple drug products. The ectoparasiticidal isoxazoline lotilaner is present in a recently marketed monodrug product formulation (Credelio™). Milbemycin oxime and lotilaner target larval and/or adult stages of various canine nematodes and major tick and flea species, respectively. Thus, Credelio Plus provides practitioners and dog owners with a straightforward, reliable and modern strategy to protect their pets from parasites. This collection of Parasites & Vectors contains articles describing detailed studies carried out in Europe and the USA to investigate the safety and effectiveness of Credelio Plus developed by Elanco for the treatment and control of major intestinal (adult and larval *Toxocara canis* and *Ancylostoma caninum*, adult *Toxascaris leonina* and *Trichuris vulpis*) and non-intestinal (*Dirofilaria immitis* and *Angiostrongylus vasorum*) nematodes and geographically important fleas (e.g. *Ctenocephalides felis* and *Ctenocephalides canis*) and ticks (e.g. *Rhipicephalus sanguineus *sensu lato, *Dermacentor reticulatus*, *Ixodes ricinus* and *Ixodes hexagonus*).

The target animal safety studies described in this article collection [1] have corroborated the historical safety data available for both milbemycin oxime and lotilaner when administered as stand-alone or combination drug products. Analogously, the acute target animal safety study showed no serious adverse events or remarkable clinical observations at the highest dosage level evaluated, i.e. six times the recommended dose of Credelio Plus [1]. The effectiveness studies confirmed the safety of Credelio Plus under both experimental and natural conditions. As outlined below, the pivotal studies that were conducted provided outstanding results against larval stages of key intestinal nematodes in controlled conditions [2, 3] and for endo- and ectoparasites in field scenarios [4–6].

*Toxocara canis* (roundworm) and *Ancylostoma caninum* (hookworm) are the most important nematodes infecting the small intestine of dogs worldwide. Dogs may become infected via various routes of transmission and both nematodes are zoonotic, i.e. *T. canis* and *A. caninum* cause *larva migrans* syndromes and skin conditions in people, respectively [7]. Although not zoonotic, *Trichuris vulpis* (whipworm) infections have a high clinical relevance in veterinary medicine. *Trichuris vulpis* is distributed worldwide, and its eggs are extremely resistant in the environment and a continuous cause of re-infections [8].

The experimental studies published in this article collection have demonstrated that the novel systemic endectocide Credelio Plus is highly effective (≥ 96.8%) against larval *T. canis* (L4) and *A. caninum* (immature adult L5) [2, 3]. These data are excellent, as endogenous larval *A. caninum* is the dose-limiting intestinal nematode for milbemycin oxime. The high efficacy of Credelio Plus against adult intestinal nematodes was confirmed by a multi-centre field study conducted in several veterinary practices in three European countries. In particular, the product proved to be highly efficacious (≥ 97.2%) in reducing the faecal egg count in dogs naturally infected with *T. canis*, *A. caninum* and *T. vulpis* [4].

Roundworms (ascarids) and hookworms are highly pathogenic, they cause severe clinical signs at both larval and adult stages and especially immature blood-sucking *A. caninum* attached to the gut mucosa may be fatal for puppies [7, 9]. The control of immature and mature *T. canis* and *A. caninum* in infected dogs is crucial. At the same time effectively treating whipworms is of great importance because *T. vulpis* causes acute or chronic large bowel inflammatory disease and severe infections are potentially lethal if not treated [8].

The canine heartworm, *D. immitis,* affects dogs in various regions of Europe, the Americas and Asia and it has some zoonotic potential. In dogs the disease is most often chronic and life-threatening and requires a challenging and health-risky medical adulticide treatment or surgical removal [10]. Thus, the prevention of *D. immitis* infections with drug substances effective against heartworm larvae is the best control approach in dogs exposed to infected mosquitoes and to reduce the risk for people living in endemic areas.

The field trial study in client-owned dogs conducted in the US and described in this article collection demonstrated outstanding effectiveness (100%) of Credelio Plus in preventing heartworm disease when administered monthly for 11 consecutive months. In fact, none of the enrolled client-owned dogs, including those living in areas historically endemic for *D. immitis*, had a heartworm infection at the end of the trial after an 11-month protection period with Credelio Plus [5]. The 100% prevention of heartworm disease by Credelio Plus is of great importance considering that many LOE/resistant isolates of *D. immitis* have been described from regions where this field study was conducted [5]. This large multi-site field study additionally provided data on the safety of Credelio Plus in a long-term clinical setting, because no treated dogs had treatment-related adverse reactions or significant laboratory parameter alterations, and there were no negative interactions between the product and medications and vaccines commonly used in canine clinical practice.

Fleas and ixodid ticks are the most important ectoparasites of dogs. They cause blood loss, discomfort, skin manifestations, allergic (fleas) and neurological (ticks) diseases, and many species are vectors of various pathogens, some with zoonotic potential [11–14].

*Ctenocephalides felis* and *Ctenocephalides canis* fleas affect dogs worldwide and both are common in Europe; these flea species may also feed on cats, other animals and humans. The presence of an infested dog implies a high contamination level of the pet’s home environment. Humidity and temperature of domestic habitats allow survival and high reproduction rates of fleas [15]. The most important ticks feeding on dogs are the “brown dog tick” *Rhipicephalus sanguineus* (*s.l.*), extremely common in urbanized habitats (including homes), and tick species belonging to the *Ixodes* and *Dermacentor* genera, which are more common in sylvatic or periurban settings [11, 12, 16].

Effective and robust control strategies for fleas and ticks are a priority in veterinary medicine and for public health issues. Especially in the case of flea infestations, control strategies should rely on an owner’s compliance because of the massive presence of environmental stages and frequent cohabitation or contact with other flea-carrier pets in the same home [15]. The concern for health issues raised by fleas and ticks is highlighted by drivers potentially fostering a geographical expansion of key species in many regions of the globe [17–19] and suspected or confirmed reduced efficacy of some molecules against certain strains [20–22].

Ectoparasiticides are administered to kill adult and/or immature stages on dogs with pre-existing infestations and/or to prevent newly acquired infestations. Products may be efficacious against adult and/or immature stages on the host and/or in their environment [13].

The field study on fleas and ticks, conducted over a period of 3 months, described in this collection has demonstrated that Credelio Plus given to client-owned dogs every 4 weeks is safe and efficacious against natural infestations by the most important species of fleas or ticks feeding on dogs, including *R. sanguineus* (*s.l.*) [6]. Importantly, excellent results from the clinical field study carried out in three European countries provided evidence to confirm the absence of isoxazoline-resistant flea and tick strains in these different geographical regions. This field trial also confirmed that dogs suffering from flea allergic dermatitis (FAD) may be greatly benefited by the administration of Credelio Plus, as previously shown by field trials conducted in the USA and in Europe using the monoproduct with lotilaner [23, 24]. The continuous month-long efficacy of Credelio Plus implies a preventive activity against flea re-infestation. This is critical to minimize FAD-induced skin lesions, as even a few bites by newly acquired fleas may cause the recurrence of clinical signs [25].

Importantly, rapid and persistent effectiveness has the potential to reduce the risk of transmission of pathogens, such as tick-borne haemoprotoza (e.g. *Babesia vogeli*) and bacteria (e.g. *Ehrlichia canis*) or flea-borne tapeworm infections (*Dipylidium caninum*).

Overall, these studies as presented in this article collection have shown the excellent palatability of Credelio Plus, as the chewable tablets were readily accepted by the vast majority of dogs (> 80% free choice, i.e. offered in empty food bowl or by hand). This characteristic is of importance as lack of compliance is a substantial hindrance to and impacts long-term control of some canine parasites (e.g. heartworms and fleas). Thus, the shortcomings inherent with the use of non-palatable products or products having a different route of administration may be easily overcome.

In summary, Credelio Plus is a palatable orally administered broad-spectrum endectocide for use in dogs with or at risk of mixed infestations/infections caused by endo- and ectoparasitic species that have the capacity to compromise the health of dogs and, in some cases, people. Dog populations living under certain epizootiological scenarios are prone to harbouring more of the above-mentioned parasites at the same time. In fact, a field study described in this article collection proved that about 30% of dogs infected by intestinal nematodes may have fleas and/or ticks as well [4], and this high prevalence of mixed parasites has also been recorded in recent epizootiological surveys from Europe and overseas [26–28]. Thus, the availability of broad-spectrum palatable oral formulations like Credelio Plus is of utmost importance where major canine parasites live in sympatry and are simultaneously endemic.

## References

[1] Riggs KL, Wiseman S (2021). Long-term and acute safety of a novel orally administered combination drug product containing milbemycin oxime and lotilaner (Credelio® Plus) in juvenile and adult dogs. Parasit Vectors.

[2] Young LM, Wiseman S, Crawley E, Bowman DD, Reinemeyer CR, Snyder DE (2021). Effectiveness of Credelio® Plus, a novel chewable tablet containing milbemycin oxime and lotilaner for the treatment of larval and immature adult stages of *Toxocara canis* in experimentally infected dogs. Parasit Vectors.

[3] Snyder DE, Wiseman S, Crawley E, Wallace K, Bowman DD, Reinemeyer CR (2021). Effectiveness of a novel orally administered combination drug product containing milbemycin oxime and lotilaner (Credelio® Plus) for the treatment of larval and immature adult stages of *Ancylostoma caninum* in experimentally infected dogs. Parasit Vectors.

[4] Hayes B, Wiseman S, Snyder DE (2021). Field study to investigate the effectiveness and safety of a novel orally administered combination drug product containing milbemycin oxime and lotilaner (Credelio® Plus) against natural intestinal nematode infections in dogs presented as veterinary patients in Europe. Parasit Vectors.

[5] Young LM, Wiseman S, Crawley E, Wallace K, Snyder DE (2021). Field study to investigate the effectiveness and safety of a novel orally administered combination drug product containing milbemycin oxime and lotilaner (Credelio® Plus) for the prevention of heartworm disease (*Dirofilaria immitis*) in client-owned dogs in the USA. Parasit Vectors.

[6] Forster S, Wiseman S, Snyder E, Wallace K, Snyder DE (2021). Field study to investigate the effectiveness and safety of a novel orally administered combination drug product containing milbemycin oxime and lotilaner (Credelio® Plus) against natural flea and tick infestations on dogs presented as veterinary patients in Europe. Parasit Vectors.

[7] Traversa D (2012). Pet roundworms and hookworms: a continuing need for global worming. Parasit Vectors.

[8] Traversa D (2011). Are we paying too much attention to cardio-pulmonary nematodes and neglecting old-fashioned worms like *Trichuris vulpis*?. Parasit Vectors.

[9] Kalkofen UP (1987). Hookworms of dogs and cats. Vet Clin North Am Small Anim Pract.

[10] McCall JW, Genchi C, Kramer LH, Guerrero J, Venco L (2008). Heartworm disease in animals and humans. Adv Parasitol.

[11] Taylor MA, Coop RL, Wall RL (2007). Veterinary parasitology.

[12] Otranto D, Dantas-Torres F (2010). Canine and feline vector-borne diseases in Italy: current situation and perspectives. Parasit Vectors.

[13] Beugnet F, Franc M (2012). Insecticide and acaricide molecules and/or combinations to prevent pet infestation by ectoparasites. Trends Parasitol.

[14] Geurden T, Becskei C, Six RH, Maeder S, Latrofa MS, Otranto D, Farkas R (2018). Detection of tick-borne pathogens in ticks from dogs and cats in different European countries. Ticks Tick Borne Dis.

[15] Traversa D (2013). Fleas infesting pets in the era of emerging extra-intestinal nematodes. Parasit Vectors.

[16] Dryden MW, Payne PA (2004). Biology and control of ticks infesting dogs and cats in North America. Vet Ther.

[17] Gray JS, Dautel H, Estrada-Peña A, Kahl O, Lindgren E (2009). Effects of climate change on ticks and tick-borne diseases in Europe. Interdiscip Perspect Infect Dis..

[18] Beugnet F, Chalvet-Monfray K (2013). Impact of climate change in the epidemiology of vector-borne diseases in domestic carnivores. Comp Immunol Microbiol Infect Dis.

[19] Medlock JM, Hansford KM, Bormane A, Derdakova M, Estrada-Peña A, George JC, Golovljova I, Jaenson TGT, Jensen JK, Jensen PM, Kazimirova M, Oteo JA, Papa A, Pfister K, Plantard O, Randolph SE, Rizzoli A, Santos-Silva MM, Sprong H, Vial L, Hendrickx G, Zeller H, Van Bortel W (2013). Driving forces for changes in geographical distribution of Ixodes ricinus ticks in Europe. Parasit Vectors.

[20] Becker S, Webster A, Doyle RL, Martins JR, Reck J, Klafke GM (2019). Resistance to deltamethrin, fipronil and ivermectin in the brown dog tick, *Rhipicephalus sanguineus* sensu stricto, Latreille (Acari: Ixodidae). Ticks Tick Borne Dis..

[21] Coles TB, Dryden MW (2014). Insecticide/acaricide resistance in fleas and ticks infesting dogs and cats. Parasit Vectors.

[22] Rust MK (2016). Insecticide resistance in fleas. Insects.

[23] Cavalleri D, Murphy M, Seewald W, Drake J, Nanchen S (2017). A randomised, blinded, controlled field study to assess the efficacy and safety of lotilaner tablets (Credelio^TM^) in controlling fleas in client-owned dogs in European countries. Parasit Vectors.

[24] Karadzovska D, Chappell K, Coble S, Murphy M, Cavalleri D, Wiseman S, Drake J, Nanchen S (2017). A randomized, controlled field study to assess the efficacy and safety of lotilaner flavored chewable tablets (Credelio^TM^) in eliminating fleas in client-owned dogs in the USA. Parasit Vectors.

[25] Carlotti DN, Jacobs DE (2000). Therapy, control and prevention of flea allergy dermatitis in dogs and cats. Vet Dermatol.

[26] Diakou A, Di Cesare A, Morelli S, Colombo M, Halos L, Simonato G, Tamvakis A, Beugnet F, Paoletti B, Traversa D (2019). Endoparasites and vector-borne pathogens in dogs from Greek islands: pathogen distribution and zoonotic implications. PLoS Negl Trop Dis.

[27] Heukelbach J, Frank R, Ariza L (2012). High prevalence of intestinal infections and ectoparasites in dogs, Minas Gerais State (southeast Brazil). Parasitol Res.

[28] Traversa D, Di Cesare A, Simonato G, Cassini R, Merola C, Diakou A, Halos L, Beugnet F, Frangipane di Regalbono A (2017). Zoonotic intestinal parasites and vector-borne pathogens in italian shelter and kennel dogs. Comp Immunol Microbiol Infect Dis.

